# 中国多发性骨髓瘤骨病诊治指南（2022年版）

**DOI:** 10.3760/cma.j.issn.0253-2727.2022.12.002

**Published:** 2022-12

**Authors:** 中国抗癌协会血液肿瘤专业委员会 中华医学会血液学分会

一、概述

多发性骨髓瘤（multiple myeloma, MM）骨病（MM bone diseases, MBD）是MM的特征性表现之一，初诊MM患者中高达80％存在溶骨性损害。2014年国际骨髓瘤工作组（IMWG）更新了MM的诊断标准，其中MBD的定义是：骨放射学检查（X线、CT、低剂量CT、MRI或PET-CT）上可见一个或一个以上穿凿样溶骨性骨破坏病灶（≥5 mm）[1]。这些患者发生骨相关事件（skeletal-related events，SRE，包括病理性骨折、脊髓压迫等）的风险极高。MBD严重影响MM患者的生活质量和生存。中华医学会血液学分会与中国抗癌协会血液肿瘤专业委员会于2011年制定了《多发性骨髓瘤骨病诊治指南（2011版）》[2]；国际骨髓瘤基金会中国多发性骨髓瘤工作组外科治疗专家委员会2016年制定了《多发性骨髓瘤骨病外科治疗中国专家共识》[3]，有力地推动了我国MBD的规范诊疗。近年来，随着MBD领域的发展，特别是靶向NF-κB配体的受体激活子（receptor activator of NF-κB Ligand，RANKL）的单克隆抗体地舒单抗的临床应用，优化了MBD的治疗[4]。为此，我们对原指南的相应内容进行了更新，以更好地指导MBD诊疗的临床实践。

二、MBD临床表现及SRE

MBD的临床特征为骨痛，常为疾病的首发症状和患者就医的主要原因之一，1/2～2/3的MM患者因骨痛就诊。骨痛部位以腰骶部最为常见，其次是胸背部。早期疼痛较轻，可为游走性或间歇性，易被误认为“风湿痛”、神经痛；后期疼痛较剧烈，活动、负重或咳嗽时加重。部分患者可无骨痛症状，仅在骨骼X线摄片时发现有骨质破坏。骨髓瘤细胞骨质浸润严重时还可形成局部肿块和骨骼变形。骨质破坏可发生在疾病的整个病程中，可导致多种SRE。SRE是指骨侵犯、骨破坏或骨损伤所致的病理性骨折、脊髓压迫、高钙血症，以及为缓解骨疼痛需要进行放射治疗和为预防或治疗需进行手术的脊髓压迫或病理性骨折等。骨髓瘤所致骨骼病变及SRE不仅严重影响患者自主活动能力和生活质量，甚至会缩短患者生存时间[5]。

三、MBD的病理生理机制

MBD的发生是骨髓瘤细胞与骨细胞、成骨细胞及破骨细胞相互作用，最终导致骨代谢和骨重塑失衡的结果。其中，RANKL/RANK/OPG（osteoprotegerin，护骨素）通路是调节骨重塑的关键通路。一方面，骨髓瘤细胞和骨髓基质细胞过度分泌RANKL，过多的RANKL与破骨细胞前体上的RANK受体结合后，促进破骨细胞的募集、分化及激活。另一方面，骨髓瘤细胞分泌dickkopf-1（DKK1）等细胞因子，骨髓基质细胞（BMSC）及成骨细胞OPG表达抑制，抑制成骨细胞功能。此外，破骨细胞降解骨并释放刺激骨髓瘤生长的因子，促进骨髓瘤细胞生长。骨髓瘤细胞与骨髓微环境相互作用，最终造成骨破坏[6]–[9]。对MBD机制的深入研究也促进了新药的研发[10]–[11]。

四、MBD的诊断

MBD的诊断主要依据影像学检查[12]。全身骨扫描应用锝［^99m^Tc］亚甲基二膦酸盐注射液（^99m^Tc-MDP），主要反映成骨细胞功能，已经不再推荐用于MBD的评估。对于头颅、四肢长骨的MBD，目前X线仍是首选。全身低剂量CT扫描可早期发现骨皮质破坏及新发的溶骨性病变。PET-CT及全身MRI作为功能性影像检查也越来越多地用于MBD的评价。这些检查各有优缺点。

1. 全身X线检查：过去广泛用于MBD的诊断评估。但其敏感性欠佳，当骨量丢失达50％～75％时才能检测到病变。四肢长骨部位的病变检出敏感性与CT相比无明显差别[13]。X线检查价格低廉、可及性高。对于头颅、四肢长骨的MBD，目前X线仍可作为首选检查。

2. CT扫描：全身低剂量CT扫描（3.2～4.8 mSv）可早期发现骨皮质破坏及新发的溶骨性病变，尤其对脊柱和骨盆病变的敏感性明显高于X线。肋骨CT三维成像有助于发现肋骨病变[14]。但CT对骨髓的早期浸润敏感性低，不能区分陈旧骨质破坏病变部位是否存在活动性骨髓瘤病变。

3. MRI检查：全身MRI（whole body MRI，WB-MRI）无电离辐射，可一次全身大范围成像，是目前最敏感的骨髓成像技术[15]–[16]，也是评估MM骨髓浸润的金标准。WB-MRI在可疑溶骨或骨质疏松部位是否存在骨髓浸润的诊断上具有重要意义。IMWG推荐所有骨髓瘤患者可应用WB-MRI检查。对于冒烟型骨髓瘤（smoldering multiple myeloma, SMM）和意义未明的单克隆免疫球蛋白增多症（monoclonal gammopathy of undetermined significance, MGUS）患者，需常规行WB-MRI检查。在MM的诊断、治疗反应[17]、微小残留病（MRD）[18]、预后评估[19]中，MRI均有重要的指导价值。扩散加权序列（DWI）和水脂分离序列（Dixon）可以反映MBD的骨髓细胞密度、组织水分和脂肪含量的细微变化[20]。表观扩散系数（apparent diffusion coefficient, ADC）和脂肪分数（fat fraction，FF）可定量评估MM患者不同的骨髓浸润模式[21]。

4. PET-CT检查：PET检查可早期发现全身活动性病灶，是检测MM伴骨骼破坏的良好手段，也可评估髓外疾病及MRD。

五、MBD的鉴别诊断

当影像学发现全身多发溶骨性病变时需注意与其他肿瘤骨转移相鉴别。转移性骨肿瘤往往可以找到原发病灶，常伴有血清碱性磷酸酶（ALP）升高，影像学显示溶骨病灶和成骨形成同时存在，局部活检有助于明确诊断。对于高钙血症，按照甲状旁腺素（PTH）是否升高可与原发性甲状旁腺亢进或其他骨转移瘤、其他血液疾病等鉴别。同时注意与骨质疏松症的鉴别诊断。

六、MBD的治疗

MBD治疗的目标包括：①缓解疼痛，降低血钙，改善生活质量；②治疗和预防SRE；③控制肿瘤进展，延长生存期。

原发病的规范化整体治疗是MBD治疗的基石[22]–[23]。通过有效的抗骨髓瘤治疗，可以阻断或延缓MM的病理进程，避免骨质破坏的进一步加重，起到治疗骨病的作用。针对MBD的主要治疗药物包括：双膦酸盐和RANKL的人源化单克隆抗体——地舒单抗。

1. 一般治疗：除非脊柱骨折的急性期，一般不建议患者绝对卧床，否则更容易发生钙丢失，应鼓励患者进行适当的活动，如步行和游泳等，但应避免剧烈运动或对抗性运动。有脊柱病变的患者应使用加有软垫的硬板床。

2. 双膦酸盐：

（1）作用原理：双膦酸盐是焦磷酸盐分子的稳定类似物。破骨细胞聚集于矿化骨基质后，通过酶水解作用溶骨。在其骨吸收过程中，双膦酸盐与羟基磷灰石晶体的暴露部分结合。破骨细胞内吞双膦酸盐，后者是细胞内法尼基焦磷酸合成酶的强抑制剂，可诱导破骨细胞凋亡，抑制溶骨[24]。

（2）适应证：所有接受抗骨髓瘤治疗的MM患者，无论影像学检查是否存在溶骨性病变，均应使用双膦酸盐。对于骨髓瘤相关的高钙血症，唑来膦酸优于帕米膦酸[25]。

对于MGUS、SMM、孤立性浆细胞瘤患者，仅在伴有骨质疏松的情况下推荐双膦酸盐，双膦酸盐的应用方法参照骨质疏松指南[26]。这些患者需要应用高敏的成像技术（包括全身低剂量CT、全身MRI，必要时PET-CT）以除外活动性MM。孤立性浆细胞瘤的治疗主要是放疗。如果放疗失败，需要按照活动性MM进行治疗，双膦酸酸盐的推荐则参照MM。对于高危的SMM，或SMM伴MRI或PET-CT的单个局限性损害，或在全身低剂量CT上有阳性发现（如在全身低剂量CT发现<5 mm的溶骨性损害，或MRI发现两个小的局限性损害），需要考虑应用双膦酸盐治疗。治疗的周期及疗程与活动性MM相同。

（3）双膦酸盐的选择、给药方式：由于双膦酸盐化学结构中与中心碳原子连接的侧链不同，因此双膦酸盐类药物的临床活性和功效亦有所不同。第一代双膦酸盐以氯膦酸二钠为代表；第二代是含氮的双膦酸盐，以帕米膦酸、阿仑膦酸钠为代表；第三代是含氮杂环结构的双膦酸盐，代表药物为唑来膦酸。唑来膦酸由于其特有的含氮杂环结构，在用药方法和疗效上均有优势。对于活动性MM，推荐应用双膦酸盐预防SRE，每3～4周给药1次。需要注意的是，无论是诊断时还是在治疗过程中都需要根据患者的肾功能调整双膦酸盐的剂量。常用双膦酸盐的剂量和用法见表1。

**表1:** 常用双膦酸盐的推荐用法

种类	用法	剂量	给药时间	肾功能不全患者用法用量调整
氯屈膦酸	口服或静脉	1 600 mg/d	口服，服药后2 h内不进饮食	SCr>442 µmol/L（5 mg/dl）时不建议使用；
				口服剂型：①CrCl 30～50 ml/min：使用常规剂量75%；②CrCl<30 ml/min：使用常规剂量50%；
				静脉剂型：①CrCl>50～80 ml/min：使用常规剂量75%～100%；②CrCl 12～50 ml/min：使用常规剂量50%～75%；③CrCl<12 ml/min：使用常规剂量50%
帕米膦酸	静脉	90 mg/月	持续至少4 h	CrCl<30 ml/min或SCr>265.2 µmol/L（3 mg/dl）时输注时间需延长至4～6 h
唑来膦酸	静脉	4 mg/月	持续至少15 min	①CrCl 50～60 ml/min：3.5 mg/月；②CrCl 40～<50 ml/min：3.3 mg/月；③CrCl 30～<40 ml/min：3 mg/月；④CrCl<30 ml/min：不建议使用

注 SCr：血肌酐；CrCl：内生肌酐清除率

与帕米膦酸相比，唑来膦酸具有更方便的给药方式。与氯屈膦酸相比，唑来膦酸在降低SRE及改善生存方面均有优势[27]。与安慰剂或者空白对照相比，仅唑来膦酸组的无进展生存（PFS）和总生存（OS）获益[28]。对于门诊及家庭给药的患者，也优先推荐唑来膦酸。

（4）疗程：从MM初始治疗即开始使用双膦酸盐。每月应用唑来膦酸，至少12个月。如果12个月后获得≥非常好的部分缓解（VGPR）的疗效，可以减少给药频率为每2～3个月1次，或者根据骨病严重程度适当延长给药间隔。如果12个月时仍未达到VGPR，则需持续每月应用唑来膦酸直至疗效≥VGPR，然后可以考虑降低给药频率或停用。停用唑来膦酸前需全面评估发生骨折的风险，包括性别、年龄、人种、体重指数、骨密度、与继发性骨质疏松相关的全身疾病状态（MM以外）、每日及累积糖皮质激素剂量。帕米膦酸的应用疗程根据上述相关因素决定。当疾病出现生化复发后，需要重新开始唑来膦酸或帕米膦酸治疗以降低新的SRE风险。具体用量用法见表2。

**表2:** 抗多发性骨髓瘤骨病（MBD）药物的选择

药物	唑来膦酸	地舒单抗	帕米膦酸
适应证	首选，无论影像学检查是否存在MBD	影像学证实存在MBD，伴肾功能不全时首选	仅在首选药物不可及或存在禁忌证时
给药方式	静脉	皮下	静脉
疗程	<VGPR，每月1次；	每月1次，持续用药	临床医师根据患者的因素及疾病相关的因素来决定
	≥VGPR，每月1次用够12个月后改为每3个月或每6个月或每年1次，或者停药；出现生化复发/进展则重新启用	如果停药，则在末次给药后至少6个月应用1次唑来膦酸；或每6个月应用1次地舒单抗	
管理	唑来膦酸及帕米膦酸：每月监测内生肌酐清除率、电解质；帕米膦酸：每月监测尿白蛋白；		
	口腔健康状况：基线，以后每年1次，或出现症状时；补充钙剂及维生素D；做好患者教育		

注 VGPR：非常好的部分缓解

3. 地舒单抗：地舒单抗是针对RANKL的完全人源化且高度特异的单克隆IgG2抗体。地舒单抗模仿OPG（又称为TNFRSF11B）的生理效应，抑制RANKL与RANK的相互作用，最终减少骨吸收[5]。

（1）适应证：具有MBD证据的MM。在延迟发生第一次SRE的时间上，地舒单抗与唑来膦酸相当。对于伴有MBD的适合移植的初诊MM（NDMM），地舒单抗可延长PFS时间。对于伴有肾损害的MM，地舒单抗优于唑来膦酸。在密切监测下，即使内生肌酐清除率低于30 ml/min，仍可应用地舒单抗[29]–[30]。MM相关的高钙血症，特别是唑来膦酸难治的患者，也可应用地舒单抗[31]。对于SMM、MGUS或孤立性浆细胞瘤，仅在患者同时存在骨质疏松时推荐应用地舒单抗（60 mg皮下注射，每6个月1次）。

（2）给药方式、方案及持续时间：地舒单抗120 mg皮下注射，每月1次，持续至给药后2年。皮下注射给药方便，门诊即可完成。当给药超过24个月，且MM疗效达到≥VGPR时，可以考虑降低给药频次甚至停药。在骨髓瘤患者中尚缺乏停药与反弹效应的相关证据，基于其治疗骨质疏松的经验，目前仍然建议最后一次地舒单抗给药后至少6个月应用1次静脉双膦酸盐（如唑来膦酸）以预防潜在的反弹效应。具体用量用法见表2。

4. 其他方法（骨水泥/椎体充填扩张术、放疗及外科手术）：所有患者均应基于病史、体格检查、实验室发现及影像学检查全面评估发生SRE的风险。发生SRE高风险的NDMM患者在给予抗骨病药物的同时还要考虑早期进行外科干预。术前建议进行脊柱稳定性评分[32]。疼痛性脊柱压缩性骨折的患者推荐进行经皮椎体球囊扩张成形术（percutaneous kyphoplasty，PKP）或椎体成形术（percutaneous vertebroplasty，PVP）。脊髓压迫或病理性骨折导致的不能控制的疼痛也可以考虑手术治疗或放疗。外科手术可预防及治疗长骨病理性骨折、脊柱不稳、脊髓压迫、髓外浆细胞瘤、周围神经卡压等。由于骨髓瘤导致长骨病理性骨折，特别是对于系统性抗骨髓瘤治疗反应微小或无反应的患者，可进行临近部位的放疗。

（1）骨水泥/椎体充填扩张术：包括PVP和PKP，可有效缓解疼痛。微创手术的时机一般在椎体骨折后4～8周内，一次手术一般最多不超过3个椎体。所有的椎体都可以进行骨水泥填充术，但需根据所处脊柱节段选择不同术式[33]。

（2）手术治疗：长骨骨折、脊髓压迫或椎体不稳等需要手术治疗。发生长骨病理性骨折需要行骨折复位、病灶刮除或切除、骨水泥填充及钛板螺钉内固定术等。外科手术能够快速缓解症状，改善生活质量[34]。但由于大多数患者为NDMM，需要尽快开始全身治疗，因此需要多学科的协作。

（3）局部放疗：对于化疗和双膦酸盐治疗后仍无法缓解的顽固性疼痛、椎体不稳、即将发生的病理性骨折和脊髓压迫，可采用局部放疗，可以有效迅速缓解骨病和软组织病变的疼痛。推荐剂量为8～10 Gy/次。局部放疗可有效控制疼痛，并有可能促进骨折愈合[35]。

（4）止痛剂的使用：若患者出现严重疼痛时需选择止痛药物。止痛药的用药剂量应作为临床治疗正式记录的一部分。这些记录可以作为疼痛治疗评估的一个半定量指标。止痛需求的减少往往意味着治疗有效。处方类止痛药的应用应遵照世界卫生组织的“止痛阶梯”原则，但尽量避免使用或要小心使用非甾体类抗炎药，因为其有肾功能损害及胃肠道刺激等不良反应。

七、双膦酸盐及地舒单抗的安全性管理

双膦酸盐类药物常见不良反应包括感冒样症状、胃肠道症状（主要是口服制剂）、低钙血症、颌骨坏死、贫血、肾功能损伤、皮疹、药物热等。其中颌骨坏死和肾脏损害尤其应重视。

维生素D为钙吸收及正常骨重塑所必需。接受双膦酸盐治疗的患者在血钙水平正常后均应补充钙片及维生素D。MM患者的具体剂量参照健康老年人推荐的钙摄入量：51～70岁的男性1 000 mg/d，50岁以上女性及70岁以上男性1 200 mg/d，最大剂量2 000 mg/d。维生素D：51～70岁600 IU/d，70岁以上800 IU/d，最大剂量4 000 IU/d。伴有肾功能不全的患者补钙需密切监测肾功能。

每月监测内生肌酐清除率、电解质、尿白蛋白，并相应作出剂量调整。双膦酸盐可能诱发急性肾衰竭，因此用药过程中常规监测肾功能非常重要[36]–[37]。推荐每次使用双膦酸盐前及在用药过程中动态监测肾功能，尤其是在每次给药前、后要保持水化状态；在双膦酸盐使用过程中尽可能避免或减少使用非甾体类药物等可能损害肾功能的药物，如果必须使用非甾体类药物，应在使用双膦酸盐24 h后使用；应避免滴注过快；推荐每月监测患者肌酐清除率（CrCl），根据CrCl调整药物剂量（具体参考表1）。

接受地舒单抗治疗的患者，尤其是伴有肾功能损害的患者，均需要补充钙片及维生素D；血钙高的患者在血钙水平降至正常后开始补充。定期监测血清钙、维生素D、磷及镁的水平，以评估是否需要额外补充。

颌骨坏死（osteonecrosis of the jaw，ONJ）是一种少见但严重的双膦酸盐/地舒单抗相关不良事件。文献报道，长期使用双膦酸盐治疗的MM患者的ONJ发病率为1.8％～12.8％[38]–[40]。地舒单抗发生ONJ的风险与唑来膦酸无明显差别[41]。治疗前及治疗期间要全面评估口腔健康状况并进行必要的预防。在拔牙及任何口腔侵入性操作前后均应暂停双膦酸盐及地舒单抗，且操作前要进行抗生素的预防性治疗。发生ONJ的患者应停止使用双膦酸盐。大多数ONJ中位4（2～6）个月能够获得愈合，后续可以重新开始双膦酸盐/地舒单抗治疗[42]。对于需要接受侵入性牙科操作的MM患者，推荐6个月（操作前3个月及操作后3个月）的无双膦酸盐/地舒单抗围手术期。侵入性牙科操作前1 d至操作后3 d需要应用抗生素预防性抗感染治疗，可以选用青霉素联合或不联合β内酰胺酶抑制剂及甲硝唑[43]。

根据疼痛、骨质暴露及齿龈肿胀程度、对抗生素冲洗的反应、是否需应用清创术及静脉抗生素，将ONJ分为三期：Ⅰ期：骨暴露无临床症状无软组织感染；Ⅱ期：骨暴露有临床症状，如疼痛或肿胀，有或无软组织/骨感染；Ⅲ期：病理性骨折、骨暴露、软组织感染。

对于ONJ尚无统一的标准化治疗准则。抗生素、口腔清洗剂、暂时停用双膦酸盐/地舒单抗和去除松动的坏死骨片对一些患者有一定效果，但广泛使用的坏死组织外科切除或清创术目前证明是无效甚至有害的。Ⅰ期患者可以予保守治疗，推荐每天用0.12％葡萄糖氯己定溶液漱口，至少每3个月复查1次。Ⅱ期患者需要根据培养结果抗感染，并予止痛治疗。Ⅲ期患者因骨坏死的量较大，仅抗感染治疗难以控制，需要外科清创术/切除术减少骨坏死数量。近年来欧美国家采用臭氧治疗获得了良好的临床疗效［Ripamonti C等在35届欧洲肿瘤内科学会（ESMO）上提出臭氧治疗ONJ的有效率可达到79％］。

八、小结

有效治疗MM是控制MBD的根本和关键所在。双膦酸盐及地舒单抗是目前MBD的标准治疗。综合考虑费用、便利度、患者偏好、不良反应等多方面的因素后选择其中的一种。在获得进一步的研究数据之前，目前优选唑来膦酸；对伴有肾损害的患者优选地舒单抗。在应用抗MBD药物的同时必须注意预防肾功能损害、低钙血症及ONJ。某些特殊情况下，如脊柱骨折、难以控制的疼痛、承重骨的骨折，需要应用PKP、PVP、放疗及外科手术治疗。
